# Fasting Plasma Glucose Indicates Reversibility of the Acute Insulin Response after Short-Term Intensive Insulin Therapy in Patients with Various Duration of Type 2 Diabetes

**DOI:** 10.1155/2018/9423965

**Published:** 2018-11-15

**Authors:** Liehua Liu, Siyue Yang, Jianbin Liu, Hai Li, Juan Liu, Xiaopei Cao, Haipeng Xiao, Yanbing Li

**Affiliations:** ^1^Department of Endocrinology and Diabetes Center, The First Affiliated Hospital of Sun Yat-Sen University, Guangzhou 510080, China; ^2^Department of Radiology, Tuen Mun Hospital, 999077, Hong Kong; ^3^Centre for Eye Research Australia, University of Melbourne, East Melbourne, VIC 3002, Australia; ^4^Department of Medicine, Box Hill Hospital, Eastern Health, Box Hill, VIC 3128, Australia

## Abstract

Recovery of acute insulin response (AIR) is shown to be associated with long-term outcomes of patients with early type 2 diabetes treated with short-term intensive insulin therapy (SIIT). However, the complexity of measuring an AIR limits its utility in a real-world clinical setting. The aim of the study was to assess fasting indicators that may estimate recovery of the AIR after SIIT. We enrolled 62 patients with type 2 diabetes mellitus (T2DM) of varying disease duration who had poor glycemic control. Participants were treated with SIIT using insulin pumps to achieve near normoglycemia for 7 days. The AIR before and after the therapy were measured by intravenous glucose tolerance tests. After the therapy, AIR increased from −16.7 (−117.4, 52.4) pmol/L·min to 178.7 (31.8, 390.7) pmol/L·min (*P* < 0.001) while hyperglycemia was alleviated; this improvement was observed in all disease duration categories. AIR was almost absent when fasting plasma glucose (FPG) > 10 mmol/L, while both AIR (*R* = −0.53, *P* < 0.001) and its improvement from baseline (△AIR, *R* = −0.52, *P* < 0.001) were negatively associated with FPG after SIIT when FPG < 10 mmol/L. In multivariate analyses, FPG after SIIT and baseline fasting C peptide were independent indicators of both AIR after the therapy and ∆AIR; HDL-C after the therapy also predicted AIR after the therapy. We concluded that recovery of the AIR could be obtained in T2DM patients of varying disease duration by SIIT and it could be conveniently estimated using posttreatment fasting plasma glucose and other fasting indicators.

## 1. Introduction

Type 2 diabetes mellitus (T2DM) is characterized by the persistent decline in *β* cell function that cannot be prevented with routine hypoglycemic strategies [1, 2]. In recent years, progression of *β* cell failure has been found to be reversible by rapid clearance of glucotoxicity via intensive hypoglycemic therapies in the early stage of disease. In our serial studies and those from other centers, short-term intensive insulin therapy (SIIT) has been proven to partly reverse *β* cell dysfunction, thereby inducing drug-free remission in over 50% of patients with newly diagnosed T2DM [3–10]. The major treatment effect is restoration of first-phase insulin secretion which was assessed by the acute insulin response (AIR) in intravenous glucose tolerance test (IVGTT), an increase in which indicates long-term drug-free remission of T2DM [3, 4]. In light of the beneficial effects of SIIT, current Chinese guideline recommends its use as a first-line therapy for patients with newly diagnosed T2DM whose blood glucose is remarkably high, in order to preserve their *β* cell function and delay the disease progression [11].

It is essential to repeatedly measure *β* cell function in order to assess treatment response to SIIT. However, performing IVGTT is laborious and expensive; frequent sampling during a short period (within 10 minutes) requires skilled staff and good patient compliance. As a result, this procedure is rarely performed in large-scale clinical research or in real-world clinical practice. Notably, in large cross-sectional studies, AIR has been shown to be significantly decreased when fasting plasma glucose (FPG) exceeds 5.6–7.0 mmol/L and is virtually nonexistent in overt diabetes [12, 13], which indicates that AIR is closely related to fasting glycemic homeostasis. Thus, it is rational to search for surrogate measures of AIR that are cheaper and more convenient to be obtained while fasted to evaluate *β* cell recovery after SIIT in practice. Moreover, most studies involving the recovery of AIR by SIIT have been performed in population with newly or recently diagnosed T2DM. In patients of longer duration, how AIR will be changed by SIIT and indicators for reversibility of impaired AIR also need to be validated.

We, therefore, conducted this prospective study by applying SIIT in patients with various T2DM duration. We analyzed the relationship between fasting parameters and AIR, in order to search for more convenient surrogates of AIR to facilitate the clinical application and monitoring strategy of SIIT.

## 2. Methods

### 2.1. Participants

Sixty-two patients, aged 20–75 years, who were diagnosed with T2DM according to WHO diagnostic criteria (1999) [14] were recruited in the First Affiliated Hospital of Sun Yat-sen University. All patients demonstrated inadequate glycemic control with glycated hemoglobin A1C (HbA1c) ≥ 7.0% (53 mmol/mol) despite stable antihyperglycemic intervention for at least 3 months. Exclusion criteria were as follows: acute or severe chronic complications of diabetes, severe concomitant diseases or use of medications affecting glucose metabolism (systemic glucocorticoids, large dose of diuretics, etc.), positive test for glutamic acid decarboxylase antibody, or pregnant or breastfeeding women. The study protocol was approved by the Research Ethics Board of the Sun Yat-Sen University and registered in https://www.clinicaltrials.gov (NCT03509324). Signed informed consent was obtained from each participant.

### 2.2. Study Design

All patients were hospitalized throughout the study and guided to start lifestyle intervention. After the withdrawal of previous antihyperglycemic therapy for at least 24 hours, anthropometric indices were measured and blood samples for the FPG, HbA1c, fasting lipid profiles, fasting C peptide, and 2 h postprandial blood glucose were collected. An IVGTT was then conducted using 25 g of glucose (50 mL of 50% glucose solution) with serum samples obtained before and 1, 2, 4, 6, and 10 min after intravenous administration of glucose solution to measure insulin. The AIR was then calculated as the incremental trapezoidal area under the curve of insulin levels during IVGTT. After baseline assessment, all patients received SIIT using an insulin pump (Paradigm 712 pump, Medtronic Inc., Northridge, CA) with insulin lispro (Humalog®, Eli Lilly and Company, USA). The initial daily insulin dose was 0.5-0.6 U/kg and was divided into basal and premeal doses with a ratio of 50% : 50%. Dosages were titrated to achieve glycemic targets (fasting/premeal blood glucose 4.4~7.0 mmol/L and 2 h postprandial blood glucose (2hPG) 4.4–10 mmol/L) based on fingertip capillary blood glucose values which were measured seven times per day (before and 2 hours after three meals, at bedtime). After glycemic targets were achieved, treatment was maintained for additional 7 days and subsequently ceased after the last premeal dose (before supper) on the 7th day. No other hypoglycemic agents or lipid-lowering agents were added during SIIT. All baseline parameters were repeated after more than 10 hours postcessation of insulin infusion and an overnight fast.

### 2.3. Laboratory Measurements

HbA1c was measured by high-performance liquid chromatography (VARIANT II; Bio-Rad, Hercules, CA). Serum insulin was measured using chemiluminescence immunoassay (Access®, Beckman Coulter, California, USA). Total cholesterol and triglyceride were assayed by enzymatic colorimetric test. High-density lipoprotein cholesterol (HDL-C) and low-density lipoprotein cholesterol (LDL-C) were measured using direct enzymatic method. All assays were done in the central laboratory of the First Affiliated Hospital of Sun Yat-sen University.

The area under the curve of insulin was calculated by trapezoidal estimation. The AIR represented the incremental area above the baseline insulin level over the duration of 10 mins. Homeostasis model assessment was also applied for estimation of *β* cell function (HOMA-B) and insulin resistance (HOMA-IR).

### 2.4. Statistical Analysis

Normally distributed variables were presented as mean ± standard deviation, and nonnormally distributed variables were presented as median (interquartile range). Patients were classified into two groups according to AIR after SIIT: group A, AIR after the therapy ≥ 300 pmol/L·min and group B, AIR after the therapy ≤ 300 pmol/L·min. This AIR value was chosen because, in one of our previous study, patients who experienced long-term remission had a mean posttreatment AIR of around 300 pmol/L·min [3]. Differences between groups were assessed with an independent *t*-test/one-way ANOVA (normally distributed variables) or Kruskal-Wallis test (nonnormally distributed variables). Differences of parameters before and after treatment were assessed with paired Student's *t*-test (normally distributed variables) or paired Wilcoxon test (nonnormally distributed variables). Associations between variables were assessed using Pearson (normally distributed variable) or Spearman's correlation (nonnormally distributed variable) analysis. Multiple linear regression was performed to identify independent indicators of AIR. Stepwise logistic regression analyses were conducted with AIR after the therapy with ≥300 pmol/L·min as the outcome. Data were analyzed with the SPSS 22.0 program and GraphPad Prism version 6.0.

## 3. Results

### 3.1. Characteristics of Participants and Treatment Effects of SIIT

**Figure 1:**
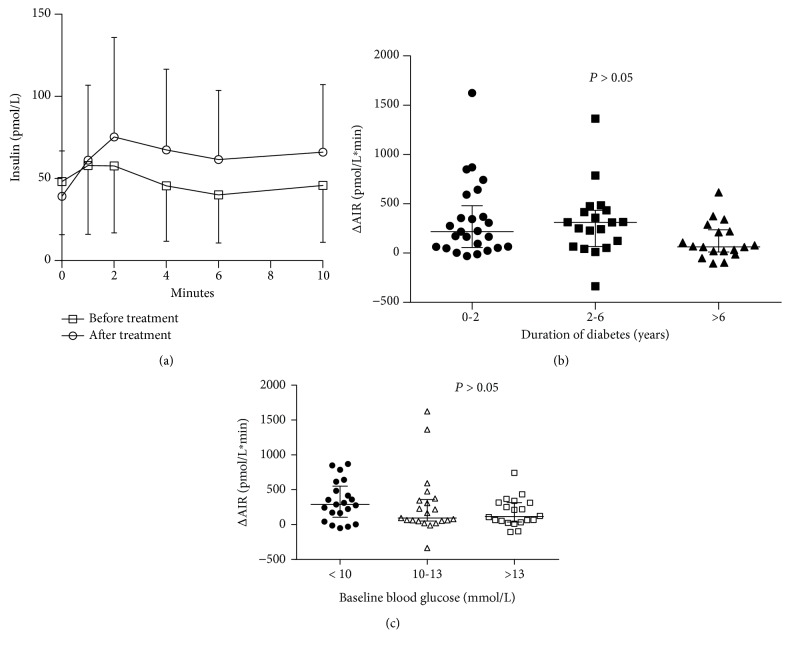
Recovery of the acute insulin response (AIR) after short-term intensive insulin therapy. The AIR significantly improved after the treatment (*P* < 0.05) (a). This effect was observed in patients with various duration of disease (b) and different baseline fasting plasma glucose levels (c).

The study population consisted of 36 men and 26 women, with a mean age of 55.9 ± 10.8 years. Median duration of diabetes was 3.0 years, and baseline HbA1c was 10.4 ± 1.9% (92.4 ± 20.0 mmol/mol). 17 (27.4%) of the participants were drug naïve, 12 (19.4%) were on oral hypoglycemic monotherapy, 26 (41.9%) were on combined oral hypoglycemic agents, and 7 (11.3%) were using insulin. The duration of insulin therapy, to achieve and maintain the predefined glycemic targets for seven days, was 12.0 ± 2.1 days. The median daily insulin dose on the first day achieving glycemic targets was 0.76 ± 0.32 U/kg. After SIIT, measures of hyperglycemia were markedly decreased: FPG (12.0 ± 3.0 mmol/L vs 7.7 ± 1.6 mmol/L, *P* < 0.001), 2 h postprandial blood glucose (17.3 ± 5.2 vs 11.5 ± 3.7 mmol/L, *P* < 0.001), and HbA1c (10.4 ± 1.9% (92.4 ± 20.0 mmol/mol) vs. 9.3 ± 1.6% (80.3 ± 16.5 mmol/mol), *P* < 0.001). Significant improvement of *β* cell function indices (AIR, −16.7 (−117.4, 52.4) pmol/L·min vs 178.7 (31.8, 390.7) pmol/L·min, *P* < 0.001; HOMA-B, 18.7 (9.6, 24.2) vs 28.5 (18.6, 42.7), *P* < 0.001) and reduction of HOMA-IR (3.4 (2.2, 5.5) vs 1.7 (1.2, 2.6), *P* < 0.001) were observed compared with baseline (Table 1 and Figure 1). Recovery of AIR was obtained in all participants across the varying duration of disease and baseline FPG categories, despite a lower but statistically insignificant treatment response in participants with longer disease duration or severe baseline hyperglycemia (Figure 1). Fasting C peptide decreased from 0.80 ± 0.29 nmol/L to 0.69 ± 0.21 nmol/L. Lipid profiles were also slightly improved (Table 1).

### 3.2. AIR Was Associated with Fasting Plasma Glucose

At baseline, participants with better AIR after therapy (group A) were younger in age, had a higher waist circumference, lower FPG, higher fasting C peptide levels, and higher HOMA-B. After SIIT, group A had significantly lower FPG, 2hPG, HbA1c, and HDL-C levels and higher fasting C peptide levels, HOMA-B, and AIR compared with group B (Table 1).

**Figure 2:**
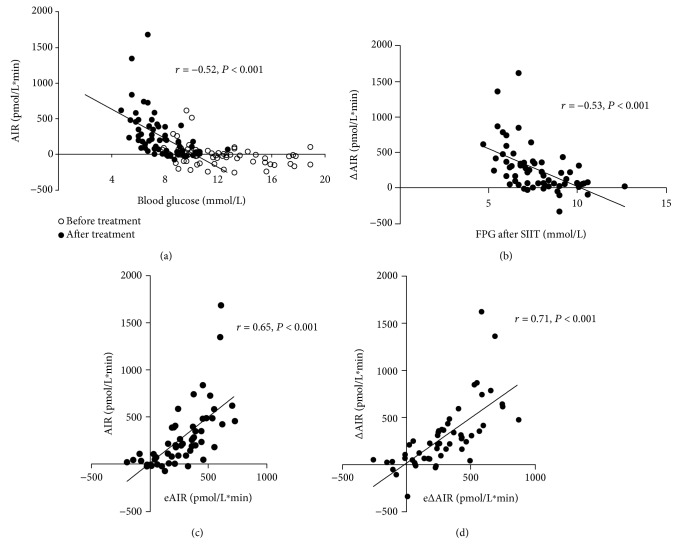
The close association between fasting plasma glucose (FPG) and acute insulin response (AIR). (a) AIR disappeared when FPG > 10 mmol/L, and it was negatively associated with FPG when FPG < 0 mmol/L. (b) FPG after SIIT was negatively correlated with ∆AIR. Performance of the estimating formulae was illustrated by comparing estimated AIR (eAIR) with actual AIR (c), as well as estimated ∆AIR (e∆AIR) with actual ∆AIR, respectively, in patients with FPG < 10 mmol/L after SIIT (*n* = 56).

To investigate the association between FPG and AIR, data before and after SIIT were pooled and plotted together. As shown in Figure 2(a), AIR was negligible with an FPG exceeding 10 mmol/L and negatively correlated with FPG when FPG was less than 10 mmol/L (*r* = −0.53, *P* < 0.001). A similar association between FPG and AIR was found at baseline and after SIIT. Improvement of AIR from baseline (△AIR) also had a negative correlation with FPG after SIIT (*r* = −0.52, *P* < 0.001, Figure 2(b)). No significant association was found between △FPG and △AIR (*r* = −0.08, *P* = 0.54).

Independent predictors of AIR recovery were explored using stepwise logistic regression models, with AIR after the therapy ≥ 300 pmol/L·min as the dependent variable. Variables with a *P* value < 0.1 in Table 1 were tested as potential candidates for independent predictors. FPG, HDL-C after SIIT, and baseline fasting serum C peptide were identified as independent predictors of higher AIR after treatment with adjustment for age, disease duration, and baseline BMI (Table 2).

### 3.3. Recovery of AIR Can Be Estimated by Fasting Biochemistry Parameters

We further developed formulae using multiple linear regression analyses in order to estimate AIR after SIIT and △AIR. As AIR was negligible if FPG exceeded 10 mmol/L (Figure 2(a)), only data from participants with FPG after SIIT less than 10 mmol/L (*n* = 56) were analyzed. In these participants, both AIR after SIIT and △AIR were normally distributed. As presented in Table 3, FPG after SIIT and baseline fasting C peptide were independently associated with both the post-SIIT AIR and △AIR. HDL-C after SIIT was also identified as an independent predictor of △AIR. Based on the models, we developed the following formulae quantitatively estimating AIR after SIIT and △AIR:

(1) $$\begin{array}{c} Estimated\;AIR\;\left(eAIR\right)\left(pmo\frac{l}{L^{*}}\min\right)=447.6\times baseline\;fasting\;C\;peptide\left(nmo\frac{l}{L}\right)-138.0\times FPG\;after\;SIIT\left(mmo\frac{l}{L}\right)+925.2,\\ Estimated\;\Delta AIR\left(e\Delta AIR\right)\left(pmo\frac{l}{L^{*}}\min\right)=401.4\times baseline\;fasting\;C\;peptide\left(nmo\frac{l}{L}\right)-136.8\times FPG\;after\;SIIT\left(mmo\frac{l}{L}\right)-357.6\times HDL\;after\;SIIT\left(mmo\frac{l}{L}\right)+1380.0. \end{array}$$

The predictive capacity of the formula was fairly good, as both the estimated eAIR and e△AIR correlated well with and were close to their respective actual values (276.8 ± 218.8 vs 280.0 ± 327.2 pmol/L^∗^min for actual AIR vs eAIR after SIIT; 286.0 ± 250.4 vs 290.2 ± 339.4 pmol/L^∗^min for △AIR vs e△AIR; both *P* > 0.05; Figures 2(c) and 2(d)).

## 4. Discussion

As expected, in this study, we demonstrated that first-phase insulin secretion was partially restored after normalizing blood glucose with SIIT in patients of varying diabetes duration. There was a robust association between posttherapy FPG and the recovery of AIR. More importantly, the improvement of AIR could be conveniently estimated using FPG and other fasting parameters, with acceptable accuracy.

AIR, which represents first-phase insulin secretion, is characterized by rapid insulin secretion shortly after (within 10 minutes) prompt elevation of blood glucose levels. Thus, AIR can significantly impact hepatic glucose output and postprandial plasma glucose excursions [15, 16]. AIR results from exocytosis of insulin granules stored within the *β* cell near plasma membrane [17, 18]. In hyperglycemia, AIR is attenuated or even lost because of a decrease in release-competent insulin granules and a disturbance in exocytosis pathways [19]. Therefore, reduced first-phase insulin secretion is present in the early stage of impaired glucose metabolism and has been well characterized as a primary defect in the development of type 2 diabetes [20].

The recovery of AIR by intensive insulin therapy has been reported in various studies over the last two decades [3–10], most of which were performed in patients with newly diagnosed T2DM. Our study, in accordance with results obtained by Stein et al. [21], suggests that *β* cell dysfunction could be reversed in participants with certain disease duration, although this beneficial treatment effect tended to attenuate in patients with longer duration of diabetes. Intensive insulin therapy facilitates *β* cell rest by normalizing blood glucose and reducing endogenous insulin demand. The reversal of *β* cell dedifferentiation (redifferentiation) [22], reduction of endoplasmic reticulum stress [23], and correction of a dysfunctional unfolded protein response could be possible explanations [24] for improved insulin production and glucose-stimulated insulin release, although a detailed mechanism still remains unclear.

There is a lack of consensus on the optimal duration of insulin therapy. In previous studies, the therapy, which usually required inpatient monitoring, lasted in varying periods, from 2 weeks to several months [25]. Our study, however, indicates that near normoglycemia for only one week was sufficient to induce significant improvement in AIR. Therefore, our findings suggesting that even a short duration of SIIT can enhance *β* cell function in patients with years of T2DM duration may extend the current indication of SIIT strategy beyond newly diagnosed T2DM [11].

Another major finding in this study is the close relationship between the recovery of AIR and simple fasting serum parameters, in particular, FPG post-SIIT. The FPG is a direct reflection of glycemic homeostasis in the basal state. Elevations of FPG from the normal range to diabetic threshold are associated with a progressive decline in *β* cell function [26, 27]; in an autopsy study, FPG showed a curvilinear relationship with relative *β* cell volume [28]. One of our previous studies revealed that lower FPG was associated with better AIR and higher long-term glycemic remission rate after SIIT in patients with newly diagnosed T2DM [29]. The other two independent predictors of AIR recovery in this study, higher fasting C peptide and lower HDL-C [30–32], are both well-recognized indicators of insulin resistance. Higher fasting C peptide also implies preserved baseline *β* cell function. These predictors are consistent with studies involving patients with newly diagnosed T2DM, in which prominent insulin resistance was associated with better treatment response to SIIT [5, 10]. Collectively, our results indicate that patients who have higher preserved baseline *β* cell function but with notable insulin resistance tend to demonstrate better recovery of AIR after SIIT, irrespective of diabetes duration and poor glycemic control.

Findings in this study also indicate the potential feasibility of a new monitoring strategy for reversibility of *β* cell function after SIIT. As mentioned previously, although AIR measurement may provide useful information for estimating glycemic outcomes after SIIT, the cost and inconvenience of the procedure limit its application. According to the study results, it is unnecessary to measure AIR if FPG exceeds 10 mmol/L as there is unlikely to be any measurable AIR. When IVGTT is infeasible, the AIR of patients with FPG less than 10 mmol/L after therapy can be estimated with reasonable predictive precision using the formulae comprising the independent predictors identified in this study.

In this study, other parameters of *β* cell function (oral challenge tests or clamp technique, etc.) were not evaluated for practical reasons. The AIR was chosen due to its sensitivity and close relationship with glycemic outcomes in previous studies concerning SIIT. Another limitation of this study is the single-arm design; however, the difference between SIIT and other therapies was not the purpose of this study. Moreover, due to the considerable size of treatment effects and the substantiation of the recovery of *β* cell function after SIIT by other studies, these treatment effects are unlikely attributed to the Hawthorne effect or chance.

In conclusion, optimal glycemic control for only one week by SIIT was sufficient to induce prominent recovery of AIR in patients of varying disease duration of T2DM and this response can be predicted and estimated using FPG after SIIT and other simple indicators. While these findings require further validation in a larger population with more diverse baseline characteristics, these discoveries may potentially result in the renewal of current practice and the development of monitoring strategies for SIIT.

## Figures and Tables

**Table 1:** Response to short-term intensive insulin therapy (SIIT) in all participants.

	Overall	Group A (*n* = 19)	Group B (*n* = 43)	*P*
Sex (male/female)	36/26	12/7	24/19	0.78
Age (years)	55.9 ± 10.8	57.3 ± 11.8	52.5 ± 7.1	0.05
Duration of diabetes (years)	3.0 (0.2, 7.3)	1.0 (0.2, 3.5)	3.5(0.3, 9.0)	0.10
Body mass index (kg/m^2^)				
Before SIIT	24.6 ± 3.0	25.7 ± 2.4	24.2 ± 3.1	0.69
After SIIT	24.6 ± 3.0	25.4 ± 2.5	24.1 ± 3.1	0.12
Waist circumference (cm)				
Before SIIT	87.4 ± 8.8	90.8 ± 7.0	85.8 ± 9.2	0.04
After SIIT	86.4 ± 8.3^∗∗^	88.9 ± 7.0^∗^	85.2 ± 8.7	0.11
Fasting plasma glucose (mmol/L)				
Before SIIT	12.0 ± 3.0	10.5 ± 2.2	12.7 ± 3.1	0.007
After SIIT	7.7 ± 1.63^∗∗^	6.6 ± 1.1^∗∗^	8.2 ± 1.6^∗∗^	<0.001
Postprandial plasma glucose (mmol/L)				
Before SIIT	17.3 ± 5.2	16.1 ± 5.2	17.8 ± 5.2	0.25
After SIIT	11.5 ± 3.7^∗∗^	9.2 ± 2.1^∗∗^	12.5 ± 3.8^∗∗^	0.001
HbA1c				
Before SIIT (%)	10.4 ± 1.9	9.9 ± 1.5	10.6 ± 2.0	0.22
(mmol/mol)	92.4 ± 20.0	87.9 ± 16.5	94.6 ± 22.0	
After SIIT (%)	9.3 ± 1.5^∗∗^	8.7 ± 1.2^∗^	9.5 ± 1.6^∗∗^	0.07
(mmol/mol)	80.3 ± 16.5 ^∗∗^	74.1 ± 13.2 ^∗^	82.5 ± 17.6 ^∗∗^	
Uric acid (*μ*mol/L)				
Before SIIT	358.4 ± 91.7	400.7 ± 68.6	339.7 ± 95.2	0.15
After SIIT	355.9 ± 78.3	383.7 ± 55.8	343.6 ± 84.0	0.03
Total cholesterol (mmol/L)				
Before SIIT	5.5 ± 1.2	5.3 ± 1.0	5.7 ± 1.3	0.27
After SIIT	5.3 ± 1.2	5.0 ± 1.1	5.4 ± 1.2	0.23
Triglyceride (mmol/L)				
Before SIIT	2.0 ± 1.3	2.1 ± 1.5	1.9 ± 1.2	0.51
After SIIT	1.5 ± 0.6^∗^	1.6 ± 0.5	1.5 ± 0.6^∗^	0.38
High-density lipoprotein cholesterol (mmol/L)				
Before SIIT	1.1 ± 0.2	1.0 ± 0.2	1.2 ± 0.3	0.15
After SIIT	1.2 ± 0.2^∗^	1.00 ± 0.2	1.3 ± 0.3^∗^	<0.001
Low-density lipoprotein cholesterol (mmol/L)				
Before SIIT	3.8 ± 0.9	3.7 ± 0.7	3.8 ± 0.9	0.46
After SIIT	3.5 ± 0.9^∗^	3.4 ± 0.8	3.6 ± 0.9^∗^	0.61
Alanine aminotransferase (U/L)				
Before SIIT	26.6 ± 14.3	28.3 ± 9.8	25.8 ± 15.9	0.53
After SIIT	26.2 ± 17.2	25.5 ± 10.3	26. 6 ± 19.6	0.82
Aspartate aminotransferase (U/L)				
Before SIIT	22.7 ± 7.5	25.0 ± 7.2	21.7 ± 7.2	0.12
After SIIT	24.7 ± 8.8^∗∗^	24.6 ± 7.0	24.8 ± 9.5^∗^	0.93
Fasting serum insulin (pmol/L)				
Before SIIT	48.0 ± 32.4	60.1 ± 31.8	43.2 ± 31.8	0.06
After SIIT	39.0 ± 27.6^∗∗^	44.9 ± 28.2^∗∗^	36.6 ± 27.6^∗^	0.27
Fasting C peptide (nmol/L)				
Before SIIT	0.8 ± 0.3	1.0 ± 0.3	0.7 ± 0.3	0.001
After SIIT	0.7 ± 0.2^∗∗^	0. 8 ± 0.2^∗∗^	0.6 ± 0.2^∗∗^	0.01
HOMA-B				
Before SIIT	18.7 (9.6, 24.2)	24.5 (16.1, 36.3)	15.5 (6.7, 23.8)	0.002
After SIIT	28.5 (18.6, 42.7)^∗∗^	49.9 (32.8, 64.9)^∗∗^	22.0 (16. 2, 32.7)^∗∗^	<0.001
HOMA-IR				
Before SIIT	3.4 (2.2, 5.5)	4.7 (2.4, 6.7)	3.1 (2.1, 4.8)	0.18
After SIIT	1.7 (1.2, 2.6)^∗∗^	1.8 (1.4, 2.4)^∗∗^	1.6 (1.2, 3.0)^∗∗^	0.77
Acute insulin response (pmol/L·min)				
Before SIIT	−16.7 (−117.4, 52.4)	16.7 ± 202.8	−23.4 ± 129.0	0.35
After SIIT	178.7 (31.8, 390.7)^∗∗^	480.0 (395.4, 725.4)^∗∗^	69.4 (5.7, 188.7)^∗∗^	<0.001
△AIR	264.5 ± 332.6	600.0 ± 390.6	116.0 ± 147.0	<0.001

Group A: participants with acute insulin response (AIR) after SIIT ≥ 300 pmol/L·min. Group B: participants with AIR after SIIT < 300 pmol/L·min. ^∗^*P* < 0.05 compared with baseline. ^∗∗^*P* < 0.001 compared with baseline.

**Table 2:** Stepwise logistic regression analysis of AIR after SIIT ≥ 300 pmol/L·min (dependent variable).

	OR	95% CI	*P*	CoxSnell *R* squared
Unadjusted model				0.47
FPG after SIIT (per mg/dL)	0.93	0.89–0.98	0.002	
HDL-C after SIIT (per mg/dL)	0.83	0.71–0.97	0.02	
Baseline fasting C peptide (per ng/mL)	3.70	1.22–11.20	0.02	
Adjusted model				
Age (>55 years)	0.41	0.05–3.04	0.38	0.50
Disease duration (>3 years)	0.44	0.07–2.69	0.38	
Baseline BMI (>25 kg/m^2^)	0.33	0.04–2.74	0.30	
FPG after SIIT (per mg/dL)	0.92	0.87–0.98	0.02	
HDL-C after SIIT (per mg/dL)	0.79	0.65–0.96	0.006	
Baseline fasting C peptide (per ng/mL)	4.67	1.21–18.02	0.03	

FPG: fasting plasma glucose; BMI: body mass index; HDL-C: high-density lipoprotein cholesterol.

**Table 3:** Multiple linear regression analysis for acute insulin response (AIR) after SIIT and change of AIR (△AIR).

	*β*	Standardized *β*	*P*	*r* ^2^	*R*_*a*_^2^
*AIR after SIIT (pmol/L·min)*					
Constant	925.2				
FPG after SIIT (mmol/L)	−138.0	−0.53	<0.001	0.42	0.39
Baseline fasting C peptide (nmol/L)	447.6	0.40	0.001		
△*AIR (pmol/L·min)*					
Constant	1380.0				
FPG after SIIT (mmol/L)	−136.8	−0.50	<0.001		
Baseline fasting C peptide (nmol/L)	401.4	0.35	0.002	0.50	0.47
HDL-C after SIIT (mmol/L)	−357.6	−0.25	0.025		

FPG: fasting plasma glucose; HDL-C: high-density lipoprotein cholesterol.

## Data Availability

The data used to support the findings of this study are available from the corresponding author upon request.
